# Ethyl 2,7,7-trimethyl-5-oxo-4-phenyl-1,4,5,6,7,8-hexa­hydro­quinoline-3-carboxyl­ate

**DOI:** 10.1107/S1600536812028371

**Published:** 2012-06-27

**Authors:** Malahat M. Kurbanova, Elnur Z. Huseynov, Atash V. Gurbanov, Abel M. Maharramov, Seik Weng Ng

**Affiliations:** a Department of Organic Chemistry, Baku State University, Baku, Azerbaijan; b Department of Chemistry, University of Malaya, 50603 Kuala Lumpur, Malaysia; c Chemistry Department, Faculty of Science, King Abdulaziz University, PO Box 80203, Jeddah, Saudi Arabia

## Abstract

In the title compound, C_21_H_25_NO_3_, the hydro­pyridine ring that constitutes a part of the hexa­hydro­quinoline fused-ring system adopts a sofa conformation; the methine C atom deviates from the least-squares plane defined by the remaining five non-H atoms (r.m.s. deviation = 0.088 Å) by 0.454 (3) Å. The phenyl ring is aligned at 85.5 (1)° with respect to this mean plane. In the crystal, adjacent molecules are linked *via* an N—H⋯O hydrogen bond, involving the amino group and the carbonyl O atom of the fused-ring system, forming chains running along [100]. The ethyl group is disordered over two positions in a 0.609 (6):0.391 (6) ratio.

## Related literature

For the synthesis, see: Safari *et al.* (2011 ▶). For the crystal structure of methyl 2,7,7-trimethyl-4-phenyl-5-oxo-1,4,5,6,7,8-hexa­hydro­quinoline-3-carboxyl­ate, see: Duque *et al.* (2000 ▶).
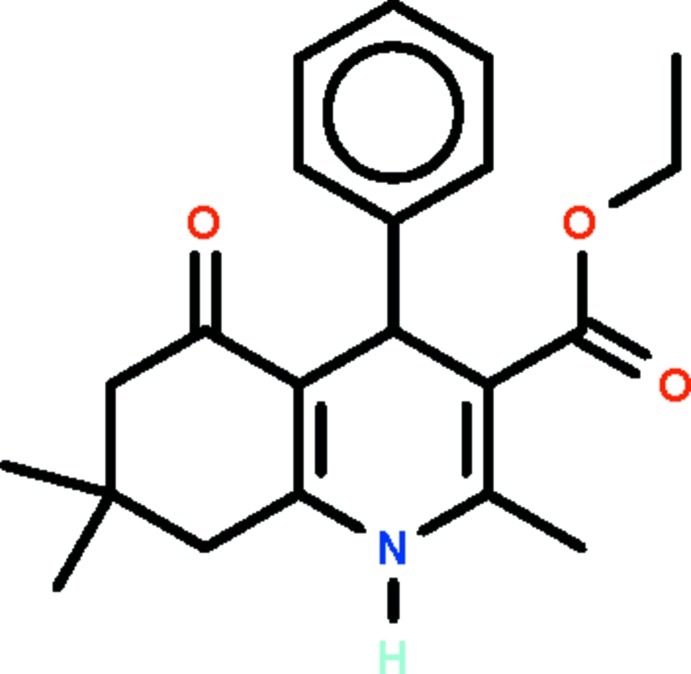


## Experimental

### 

#### Crystal data

C_21_H_25_NO_3_
*M* *_r_* = 339.42
Triclinic, 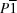

*a* = 7.3523 (4) Å
*b* = 9.6349 (5) Å
*c* = 13.9495 (7) Å
α = 98.370 (1)°
β = 91.778 (1)°
γ = 106.291 (1)°
*V* = 935.70 (8) Å^3^
*Z* = 2
Mo *K*α radiation
μ = 0.08 mm^−1^
*T* = 296 K
0.20 × 0.20 × 0.20 mm

#### Data collection

Bruker SMART APEX diffractometer
10191 measured reflections
4302 independent reflections
3439 reflections with *I* > 2σ(*I*)
*R*_int_ = 0.016

#### Refinement

*R*[*F* ^2^ > 2σ(*F* ^2^)] = 0.046
*wR*(*F* ^2^) = 0.135
*S* = 1.03
4302 reflections
254 parameters
4 restraints
H atoms treated by a mixture of independent and constrained refinement
Δρ_max_ = 0.27 e Å^−3^
Δρ_min_ = −0.20 e Å^−3^

### 

Data collection: *APEX2* (Bruker, 2005 ▶); cell refinement: *SAINT* (Bruker, 2005 ▶); data reduction: *SAINT*; program(s) used to solve structure: *SHELXS97* (Sheldrick, 2008 ▶); program(s) used to refine structure: *SHELXL97* (Sheldrick, 2008 ▶); molecular graphics: *X-SEED* (Barbour, 2001 ▶); software used to prepare material for publication: *publCIF* (Westrip, 2010 ▶).

## Supplementary Material

Crystal structure: contains datablock(s) global, I. DOI: 10.1107/S1600536812028371/xu5576sup1.cif

Structure factors: contains datablock(s) I. DOI: 10.1107/S1600536812028371/xu5576Isup2.hkl

Supplementary material file. DOI: 10.1107/S1600536812028371/xu5576Isup3.cml

Additional supplementary materials:  crystallographic information; 3D view; checkCIF report

## Figures and Tables

**Table 1:** Hydrogen-bond geometry (Å, °)

*D*—H⋯*A*	*D*—H	H⋯*A*	*D*⋯*A*	*D*—H⋯*A*
N1—H1⋯O1^i^	0.87 (1)	2.04 (1)	2.890 (1)	168 (2)

Symmetry code: (i) 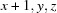.

**Fig. 1.**
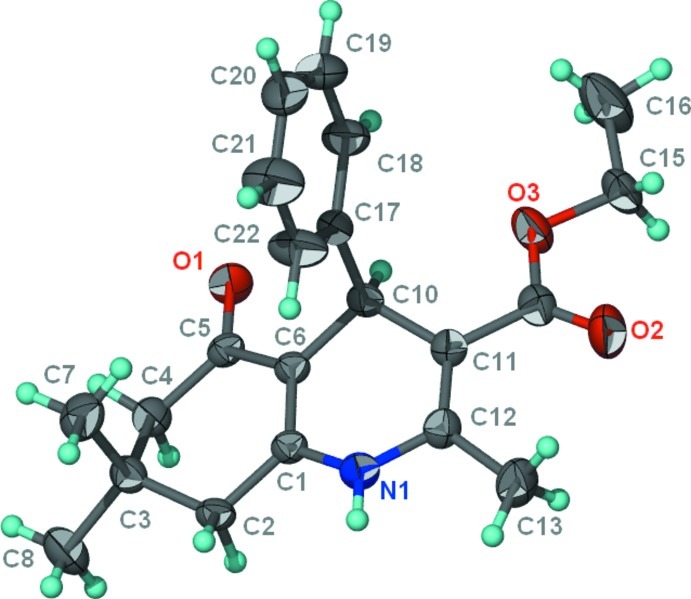
Thermal ellipsoid plot (Barbour, 2001) of C23H26O4 at the 50% probability level; H atoms are drawn as spheres of arbitrary radius. The disorder is not shown.

**Fig. 2.**
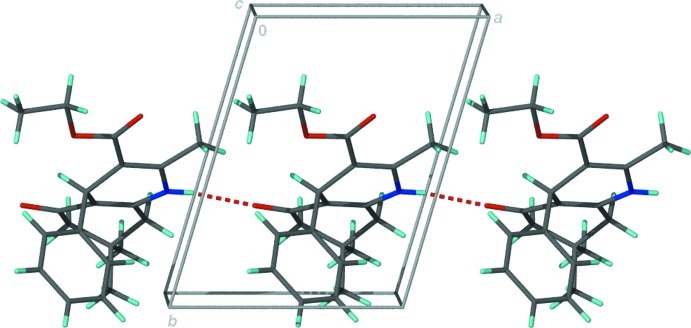
Hydrogen-bonded chain motif.

## supplementary crystallographic information

### Comment

C5-Unsubstituted 1,4-dihydropyridines are readily synthesized by the reaction of dimedone, acetophenone, aromatic aldehydes, and ammonium acetate in the presence of a catalytic amount of a cobalt salt under solvent-free conditions (Safari *et al.* 2011). The title compound (Scheme I), was synthesized by a slightly different procedure from benzaldehyde, ethyl acetoacetate and ammonium acetate and with *L*-glutamine as catalyst, and in ethanol medium.

The hydropyridine ring that constitutes a part of the hexahydroquinoline fused-ring system of C_21_H_25_NO_3_ adopts a sofa conformation; the methine C atom bearing the phenyl substituent deviates from the least-squares plane defined by the N and four double-bond C atoms by 0.454 (3) Å. The phenyl ring is nearly orthogornal to this plane (Fig. 1). The amino group is hydrogen-bond donor to the carbonyl O atom of the fused-ring of another molecule; adjacent molecules are linked by an N—H···O hydrogen bond to form a chain running along the *a*-axis of the triclinic cell (Table 1, Fig. 2).

### Experimental

Dimedone (0.56 g, 2 mmol) was stirred with benzaldehyde (0.40 ml, 2 mmol), ethyl acetoacetate (0.5 ml, 2 mmol) and ammonium acetate (0.308 g, 2 mmol) in ethanol (50 ml) for 12 h at the room temperature. A small quantity of *L*-glutamine (0.05 g) was added as catalyst. Recrystallization was effected by using ethanol as solvent.

### Refinement

Carbon-bound H atoms were placed in calculated positions [C—H 0.93 to 0.98 Å; *U*(H) 1.2 to 1.5*U*(C)] and were included in the refinement in the riding model approximation.

The amino H atom was located in a difference Fourier map and was refined with a distance restraint of N—H 0.88±0.01 Å; its temperature factor was refined.

The ethyl group is disordered over two sites in a 0.609 (6):0.391 ratio. The C—C distances were restrained to 1.54±0.01 Å.

The (0 0 1) reflection was omitted owing to bad disagreement.

### Crystal data

**Table:**

C_21_H_25_NO_3_	*Z* = 2
*M**_r_* = 339.42	*F*(000) = 364
Triclinic, *P*1	*D*_x_ = 1.205 Mg m^−^^3^
Hall symbol: -P 1	Mo *K*α radiation, λ = 0.71073 Å
*a* = 7.3523 (4) Å	Cell parameters from 4443 reflections
*b* = 9.6349 (5) Å	θ = 2.2–29.2°
*c* = 13.9495 (7) Å	µ = 0.08 mm^−^^1^
α = 98.370 (1)°	*T* = 296 K
β = 91.778 (1)°	Prism, yellow
γ = 106.291 (1)°	0.20 × 0.20 × 0.20 mm
*V* = 935.70 (8) Å^3^	

### Data collection

**Table:**

Bruker SMART APEX diffractometer	3439 reflections with *I* > 2σ(*I*)
Radiation source: fine-focus sealed tube	*R*_int_ = 0.016
Graphite monochromator	θ_max_ = 27.5°, θ_min_ = 2.2°
φ and ω scans	*h* = −9→9
10191 measured reflections	*k* = −12→12
4302 independent reflections	*l* = −17→18

### Refinement

**Table:**

Refinement on *F*^2^	Primary atom site location: structure-invariant direct methods
Least-squares matrix: full	Secondary atom site location: difference Fourier map
*R*[*F*^2^ > 2σ(*F*^2^)] = 0.046	Hydrogen site location: inferred from neighbouring sites
*wR*(*F*^2^) = 0.135	H atoms treated by a mixture of independent and constrained refinement
*S* = 1.03	*w* = 1/[σ^2^(*F*_o_^2^) + (0.0682*P*)^2^ + 0.1935*P*] where *P* = (*F*_o_^2^ + 2*F*_c_^2^)/3
4302 reflections	(Δ/σ)_max_ = 0.001
254 parameters	Δρ_max_ = 0.27 e Å^−^^3^
4 restraints	Δρ_min_ = −0.20 e Å^−^^3^

### Fractional atomic coordinates and isotropic or equivalent isotropic displacement parameters (Å^2^)

**Table:**

	*x*	*y*	*z*	*U*_iso_*/*U*_eq_	Occ. (<1)
O1	0.25249 (12)	0.66980 (12)	0.42973 (8)	0.0499 (3)	
O2	0.6353 (2)	0.34700 (15)	0.10252 (9)	0.0749 (4)	
O3	0.37322 (15)	0.41542 (13)	0.12222 (8)	0.0563 (3)	
N1	0.85485 (15)	0.61848 (13)	0.37295 (8)	0.0404 (3)	
H1	0.9683 (15)	0.6251 (18)	0.3964 (11)	0.053 (4)*	
C1	0.73337 (16)	0.66880 (13)	0.43108 (9)	0.0327 (3)	
C2	0.80247 (17)	0.72215 (15)	0.53537 (9)	0.0376 (3)	
H2A	0.9355	0.7777	0.5391	0.045*	
H2B	0.7932	0.6383	0.5679	0.045*	
C3	0.69190 (18)	0.81798 (14)	0.58871 (9)	0.0390 (3)	
C4	0.48007 (18)	0.74010 (16)	0.56507 (10)	0.0407 (3)	
H4A	0.4463	0.6554	0.5980	0.049*	
H4B	0.4088	0.8058	0.5913	0.049*	
C5	0.41906 (16)	0.69006 (13)	0.45882 (9)	0.0338 (3)	
C6	0.55737 (16)	0.66029 (13)	0.39380 (9)	0.0317 (3)	
C7	0.7417 (2)	0.96857 (16)	0.55736 (13)	0.0584 (4)	
H7A	0.7096	0.9577	0.4889	0.088*	
H7B	0.8754	1.0157	0.5711	0.088*	
H7C	0.6715	1.0271	0.5924	0.088*	
C8	0.7403 (3)	0.8357 (2)	0.69817 (11)	0.0626 (5)	
H8A	0.8733	0.8847	0.7129	0.094*	
H8B	0.7106	0.7409	0.7178	0.094*	
H8C	0.6673	0.8926	0.7324	0.094*	
C10	0.50411 (16)	0.61688 (14)	0.28579 (9)	0.0341 (3)	
H10	0.3704	0.5585	0.2768	0.041*	
C11	0.62229 (17)	0.52035 (13)	0.24136 (9)	0.0359 (3)	
C12	0.79381 (18)	0.52991 (15)	0.28380 (10)	0.0387 (3)	
C13	0.9302 (2)	0.4477 (2)	0.24766 (13)	0.0604 (4)	
H13A	0.9613	0.4664	0.1835	0.091*	
H13B	0.8727	0.3446	0.2458	0.091*	
H13C	1.0438	0.4794	0.2905	0.091*	
C14	0.5505 (2)	0.41942 (15)	0.14984 (10)	0.0436 (3)	
C15	0.3012 (10)	0.3078 (6)	0.0336 (4)	0.0601 (14)	0.609 (6)
H15A	0.2845	0.2090	0.0467	0.072*	0.609 (6)
H15B	0.3884	0.3257	−0.0170	0.072*	0.609 (6)
C16	0.1147 (5)	0.3283 (6)	0.0039 (3)	0.101 (2)	0.609 (6)
H16A	0.0707	0.2739	−0.0598	0.151*	0.609 (6)
H16B	0.1299	0.4304	0.0031	0.151*	0.609 (6)
H16C	0.0237	0.2938	0.0493	0.151*	0.609 (6)
C15'	0.2769 (16)	0.3466 (8)	0.0264 (6)	0.060 (2)	0.391 (6)
H15C	0.3674	0.3276	−0.0192	0.072*	0.391 (6)
H15D	0.2102	0.4088	0.0010	0.072*	0.391 (6)
C16'	0.1393 (9)	0.2054 (7)	0.0435 (4)	0.087 (2)	0.391 (6)
H16D	0.0707	0.1536	−0.0169	0.131*	0.391 (6)
H16E	0.0515	0.2265	0.0889	0.131*	0.391 (6)
H16F	0.2079	0.1460	0.0692	0.131*	0.391 (6)
C17	0.52448 (18)	0.74910 (14)	0.23461 (9)	0.0382 (3)	
C18	0.3791 (2)	0.75906 (18)	0.17376 (12)	0.0542 (4)	
H18	0.2641	0.6854	0.1657	0.065*	
C19	0.4008 (3)	0.8766 (2)	0.12439 (13)	0.0653 (5)	
H19	0.3011	0.8801	0.0830	0.078*	
C20	0.5650 (3)	0.98628 (19)	0.13566 (12)	0.0629 (5)	
H20	0.5786	1.0659	0.1031	0.075*	
C21	0.7100 (3)	0.9784 (2)	0.19528 (16)	0.0760 (6)	
H21	0.8239	1.0531	0.2032	0.091*	
C22	0.6909 (2)	0.86116 (19)	0.24438 (14)	0.0644 (5)	
H22	0.7923	0.8580	0.2847	0.077*	

### Atomic displacement parameters (Å^2^)

**Table:**

	*U*^11^	*U*^22^	*U*^33^	*U*^12^	*U*^13^	*U*^23^
O1	0.0253 (4)	0.0631 (7)	0.0601 (6)	0.0167 (4)	−0.0015 (4)	−0.0010 (5)
O2	0.0783 (9)	0.0794 (9)	0.0642 (8)	0.0368 (7)	−0.0039 (6)	−0.0231 (6)
O3	0.0480 (6)	0.0667 (7)	0.0437 (6)	0.0101 (5)	−0.0078 (5)	−0.0091 (5)
N1	0.0226 (5)	0.0514 (7)	0.0460 (6)	0.0130 (4)	−0.0010 (4)	0.0002 (5)
C1	0.0254 (5)	0.0343 (6)	0.0382 (6)	0.0086 (4)	0.0010 (5)	0.0057 (5)
C2	0.0292 (6)	0.0431 (7)	0.0403 (7)	0.0108 (5)	−0.0047 (5)	0.0064 (5)
C3	0.0364 (6)	0.0404 (7)	0.0377 (7)	0.0093 (5)	−0.0008 (5)	0.0026 (5)
C4	0.0344 (6)	0.0476 (7)	0.0406 (7)	0.0126 (5)	0.0070 (5)	0.0061 (6)
C5	0.0269 (6)	0.0313 (6)	0.0438 (7)	0.0090 (4)	0.0020 (5)	0.0070 (5)
C6	0.0262 (5)	0.0333 (6)	0.0358 (6)	0.0090 (4)	0.0006 (4)	0.0056 (5)
C7	0.0579 (9)	0.0381 (8)	0.0743 (11)	0.0086 (7)	−0.0004 (8)	0.0051 (7)
C8	0.0567 (10)	0.0840 (12)	0.0411 (8)	0.0180 (9)	−0.0032 (7)	−0.0033 (8)
C10	0.0247 (5)	0.0388 (6)	0.0367 (6)	0.0077 (5)	−0.0022 (4)	0.0032 (5)
C11	0.0331 (6)	0.0356 (6)	0.0377 (6)	0.0078 (5)	0.0038 (5)	0.0049 (5)
C12	0.0313 (6)	0.0409 (7)	0.0435 (7)	0.0101 (5)	0.0071 (5)	0.0046 (5)
C13	0.0422 (8)	0.0713 (11)	0.0683 (10)	0.0269 (7)	0.0059 (7)	−0.0086 (8)
C14	0.0467 (8)	0.0410 (7)	0.0402 (7)	0.0090 (6)	0.0030 (6)	0.0043 (6)
C15	0.058 (2)	0.068 (3)	0.045 (2)	0.017 (2)	−0.0148 (15)	−0.013 (2)
C16	0.058 (2)	0.160 (5)	0.068 (2)	0.033 (2)	−0.0198 (17)	−0.035 (3)
C15'	0.073 (5)	0.061 (4)	0.042 (3)	0.018 (3)	−0.011 (3)	0.002 (3)
C16'	0.083 (4)	0.086 (4)	0.066 (3)	−0.006 (3)	−0.001 (3)	−0.011 (3)
C17	0.0397 (7)	0.0417 (7)	0.0341 (6)	0.0151 (5)	−0.0006 (5)	0.0035 (5)
C18	0.0492 (8)	0.0576 (9)	0.0572 (9)	0.0180 (7)	−0.0099 (7)	0.0118 (7)
C19	0.0742 (11)	0.0711 (11)	0.0596 (10)	0.0339 (10)	−0.0114 (9)	0.0177 (8)
C20	0.0906 (13)	0.0535 (9)	0.0528 (9)	0.0300 (9)	0.0043 (9)	0.0174 (7)
C21	0.0701 (12)	0.0607 (11)	0.0899 (14)	−0.0019 (9)	−0.0095 (10)	0.0331 (10)
C22	0.0498 (9)	0.0603 (10)	0.0781 (12)	0.0010 (7)	−0.0178 (8)	0.0298 (9)

### Geometric parameters (Å, º)

**Table:**

O1—C5	1.2307 (14)	C11—C12	1.3498 (18)
O2—C14	1.2023 (18)	C11—C14	1.4706 (18)
O3—C14	1.3364 (18)	C12—C13	1.4992 (19)
O3—C15'	1.466 (6)	C13—H13A	0.9600
O3—C15	1.470 (4)	C13—H13B	0.9600
N1—C1	1.3652 (16)	C13—H13C	0.9600
N1—C12	1.3848 (17)	C15—C16	1.493 (6)
N1—H1	0.868 (9)	C15—H15A	0.9700
C1—C6	1.3557 (16)	C15—H15B	0.9700
C1—C2	1.4930 (17)	C16—H16A	0.9600
C2—C3	1.5249 (18)	C16—H16B	0.9600
C2—H2A	0.9700	C16—H16C	0.9600
C2—H2B	0.9700	C15'—C16'	1.506 (9)
C3—C7	1.526 (2)	C15'—H15C	0.9700
C3—C4	1.5290 (18)	C15'—H15D	0.9700
C3—C8	1.531 (2)	C16'—H16D	0.9600
C4—C5	1.5032 (18)	C16'—H16E	0.9600
C4—H4A	0.9700	C16'—H16F	0.9600
C4—H4B	0.9700	C17—C22	1.374 (2)
C5—C6	1.4437 (17)	C17—C18	1.3775 (19)
C6—C10	1.5121 (17)	C18—C19	1.384 (2)
C7—H7A	0.9600	C18—H18	0.9300
C7—H7B	0.9600	C19—C20	1.350 (3)
C7—H7C	0.9600	C19—H19	0.9300
C8—H8A	0.9600	C20—C21	1.357 (3)
C8—H8B	0.9600	C20—H20	0.9300
C8—H8C	0.9600	C21—C22	1.381 (2)
C10—C17	1.5230 (18)	C21—H21	0.9300
C10—C11	1.5242 (17)	C22—H22	0.9300
C10—H10	0.9800		
			
C14—O3—C15'	124.4 (5)	C12—C11—C10	120.57 (11)
C14—O3—C15	110.9 (3)	C14—C11—C10	119.40 (11)
C1—N1—C12	122.25 (10)	C11—C12—N1	119.49 (11)
C1—N1—H1	119.8 (11)	C11—C12—C13	127.20 (13)
C12—N1—H1	116.6 (11)	N1—C12—C13	113.23 (12)
C6—C1—N1	119.61 (11)	C12—C13—H13A	109.5
C6—C1—C2	124.11 (11)	C12—C13—H13B	109.5
N1—C1—C2	116.17 (10)	H13A—C13—H13B	109.5
C1—C2—C3	113.38 (10)	C12—C13—H13C	109.5
C1—C2—H2A	108.9	H13A—C13—H13C	109.5
C3—C2—H2A	108.9	H13B—C13—H13C	109.5
C1—C2—H2B	108.9	O2—C14—O3	121.58 (13)
C3—C2—H2B	108.9	O2—C14—C11	126.43 (14)
H2A—C2—H2B	107.7	O3—C14—C11	112.00 (12)
C2—C3—C7	111.03 (12)	O3—C15—C16	105.3 (4)
C2—C3—C4	107.82 (10)	O3—C15—H15A	110.7
C7—C3—C4	110.04 (12)	C16—C15—H15A	110.7
C2—C3—C8	108.95 (12)	O3—C15—H15B	110.7
C7—C3—C8	109.41 (13)	C16—C15—H15B	110.7
C4—C3—C8	109.56 (12)	H15A—C15—H15B	108.8
C5—C4—C3	115.31 (11)	O3—C15'—C16'	104.8 (6)
C5—C4—H4A	108.4	O3—C15'—H15C	110.8
C3—C4—H4A	108.4	C16'—C15'—H15C	110.8
C5—C4—H4B	108.4	O3—C15'—H15D	110.8
C3—C4—H4B	108.4	C16'—C15'—H15D	110.8
H4A—C4—H4B	107.5	H15C—C15'—H15D	108.9
O1—C5—C6	121.51 (12)	C15'—C16'—H16D	109.5
O1—C5—C4	119.89 (11)	C15'—C16'—H16E	109.5
C6—C5—C4	118.53 (10)	H16D—C16'—H16E	109.5
C1—C6—C5	119.17 (11)	C15'—C16'—H16F	109.5
C1—C6—C10	120.72 (10)	H16D—C16'—H16F	109.5
C5—C6—C10	120.10 (10)	H16E—C16'—H16F	109.5
C3—C7—H7A	109.5	C22—C17—C18	117.07 (14)
C3—C7—H7B	109.5	C22—C17—C10	121.47 (12)
H7A—C7—H7B	109.5	C18—C17—C10	121.42 (12)
C3—C7—H7C	109.5	C17—C18—C19	121.25 (16)
H7A—C7—H7C	109.5	C17—C18—H18	119.4
H7B—C7—H7C	109.5	C19—C18—H18	119.4
C3—C8—H8A	109.5	C20—C19—C18	120.74 (16)
C3—C8—H8B	109.5	C20—C19—H19	119.6
H8A—C8—H8B	109.5	C18—C19—H19	119.6
C3—C8—H8C	109.5	C19—C20—C21	118.86 (16)
H8A—C8—H8C	109.5	C19—C20—H20	120.6
H8B—C8—H8C	109.5	C21—C20—H20	120.6
C6—C10—C17	112.36 (10)	C20—C21—C22	121.03 (17)
C6—C10—C11	109.62 (10)	C20—C21—H21	119.5
C17—C10—C11	111.30 (10)	C22—C21—H21	119.5
C6—C10—H10	107.8	C17—C22—C21	121.04 (15)
C17—C10—H10	107.8	C17—C22—H22	119.5
C11—C10—H10	107.8	C21—C22—H22	119.5
C12—C11—C14	120.00 (12)		
			
C12—N1—C1—C6	−14.66 (19)	C10—C11—C12—N1	5.87 (19)
C12—N1—C1—C2	161.64 (12)	C14—C11—C12—C13	0.7 (2)
C6—C1—C2—C3	−21.55 (17)	C10—C11—C12—C13	−177.44 (14)
N1—C1—C2—C3	162.33 (11)	C1—N1—C12—C11	16.3 (2)
C1—C2—C3—C7	−72.94 (14)	C1—N1—C12—C13	−160.84 (13)
C1—C2—C3—C4	47.67 (14)	C15'—O3—C14—O2	−11.4 (5)
C1—C2—C3—C8	166.50 (12)	C15—O3—C14—O2	3.4 (4)
C2—C3—C4—C5	−50.59 (15)	C15'—O3—C14—C11	168.4 (4)
C7—C3—C4—C5	70.64 (15)	C15—O3—C14—C11	−176.8 (3)
C8—C3—C4—C5	−169.02 (12)	C12—C11—C14—O2	−5.1 (2)
C3—C4—C5—O1	−157.03 (12)	C10—C11—C14—O2	172.99 (15)
C3—C4—C5—C6	25.84 (17)	C12—C11—C14—O3	175.07 (12)
N1—C1—C6—C5	169.51 (11)	C10—C11—C14—O3	−6.81 (17)
C2—C1—C6—C5	−6.49 (18)	C14—O3—C15—C16	−172.8 (4)
N1—C1—C6—C10	−9.10 (18)	C15'—O3—C15—C16	−34 (2)
C2—C1—C6—C10	174.90 (11)	C14—O3—C15'—C16'	104.6 (8)
O1—C5—C6—C1	−172.73 (12)	C15—O3—C15'—C16'	56.8 (19)
C4—C5—C6—C1	4.35 (17)	C6—C10—C17—C22	53.16 (18)
O1—C5—C6—C10	5.89 (18)	C11—C10—C17—C22	−70.21 (17)
C4—C5—C6—C10	−177.03 (11)	C6—C10—C17—C18	−129.18 (14)
C1—C6—C10—C17	−96.98 (13)	C11—C10—C17—C18	107.45 (15)
C5—C6—C10—C17	84.42 (13)	C22—C17—C18—C19	0.3 (2)
C1—C6—C10—C11	27.33 (15)	C10—C17—C18—C19	−177.41 (14)
C5—C6—C10—C11	−151.27 (11)	C17—C18—C19—C20	−0.9 (3)
C6—C10—C11—C12	−25.63 (16)	C18—C19—C20—C21	0.9 (3)
C17—C10—C11—C12	99.29 (14)	C19—C20—C21—C22	−0.4 (3)
C6—C10—C11—C14	156.26 (11)	C18—C17—C22—C21	0.2 (3)
C17—C10—C11—C14	−78.82 (14)	C10—C17—C22—C21	177.95 (17)
C14—C11—C12—N1	−176.02 (12)	C20—C21—C22—C17	−0.2 (3)

### Hydrogen-bond geometry (Å, º)

**Table:**

*D*—H···*A*	*D*—H	H···*A*	*D*···*A*	*D*—H···*A*
N1—H1···O1^i^	0.87 (1)	2.04 (1)	2.890 (1)	168 (2)

Symmetry code: (i) *x*+1, *y*, *z*.
